# Supplementary material for: WordCommentsAnalyzer: A windows software tool for qualitative research
Source: F1000Res. 2018 Sep 4;7:536. Originally published 2018 May 3. [Version 2] doi: 10.12688/f1000research.14819.2 (PMC6137411; doi:10.12688/f1000research.14819.2)
Supplement: Supplementary file 3 [file f1000research-7-17614-s0002.tgz › b2a79496-4ada-47c4-9682-a99ce867836d.zip!/Eight Iranian Health Journals/Journal of Health System Research _45.docx]

© Journal of Health System Research . This work is licensed under CC BY-NC 4.0 (https://creativecommons.org/licenses/by-nc/4.0/).

Volume & Issue: دوره 6، شماره 4؛ 1389

Title: چالش‌های کاربرد فناوری‌های نوین اطلاعاتی در دانشگاه‌های علوم پزشکی

http://hsr.mui.ac.ir/index.php/jhsr/article/view/129

مقدمه: ارائه سیستم‌های فناوری اطلاعات در یک سازمان همراه با ریسک بوده و با چالش‌های متعددی روبه‌رو است. این تحقیق با هدف بررسی موانع و چالش‌های فراروی استفاده از فناوری‌های نوین اطلاعاتی (ICT) در دانشگاه‌هاي علوم پزشكي و به صورت موردي در دانشگاه علوم پزشكي ايلام صورت گرفته است. روش‌ها: جامعه آماری این پژوهش، کلیه کارکنان دانشگاه علوم پزشکی ایلام می‌باشند که 206 نفر از آنان به عنوان نمونه انتخاب گردید. ابزار گردآوری داده‌ها، پرسش‌نامه محقق ساخته بوده است و به منظور دسته‌بندی موانع کاربرد فناوری‌‌‌های نوین از روش تحلیل عاملی اکتشافی استفاده گرديد. یافته‌ها: چالش‌های کاربرد ICT در دانشگاه‌هاي علوم پزشكي در هفت عامل خلاصه گردیدند:  1. فراهم نبودن زیرساخت سخت‌افزاری  2. عدم راهبردهای توسعه اطلاعاتی در سازمان 3. عدم دسترسی به برنامه‌های کاربردی آسان و راحت  4. عدم توانایی کارکنان در کار با نرم‌افزارها و برنامه‌های موجود  5. عدم باور مدیران 6. بالا بودن هزینه تغییر از سیستم‌های سنتی به سیستم‌های جدید  7. مقاومت کارکنان. نتیجه‌گیری: فراهم نمودن زیرساخت‌ها، تنظیم سند راهبردی توسعه اطلاعات و به كارگيري برنامه‌‌های ساده و کاربرپسند می‌تواند به پذیرش فناوری‌هاي نوين اطلاعاتي در دانشگاه کمک نماید.

Title: کاربرد مدل معادلات ساختاری در تعیین روایی سازه یک روش غربال‌گری استرین گرمایی

http://hsr.mui.ac.ir/index.php/jhsr/article/view/41

مقدمه: تنش گرمایی یکی از مهم‌ترین پیامد‌های مواجهه با گرما در دنیاست. شاخص‌های موجود ارزیابی تنش گرمایی به دلیل داشتن محدودیت‌های ذاتی و کاربردی برای غربال‌گری تنش گرمایی، در کشور‌های در حال توسعه مناسب نیستند. هدف این مطالعه بررسی روایی محتوایی و اعتبار سازه شاخص نمره تنش گرمایی (Heat Strain Score Index یا HSSI) در شرایط آب و هوایی ایران بود. روش‌ها: این مطالعه مقطعی بر روی 122 نفر از کارگران شرکت ملی صنایع پتروشیمی (عسلویه) و ذوب‌آهن اصفهان در ماه‌های تیر تا شهریور 1389 انجام شد. در مرحله تولید آیتم، 45 آیتم انتخاب گردید. روایی محتوایی توسط متخصصین بهداشت حرفه‌ای انجام گرفت و ثبات درونی آیتم‌ها با محاسبه ضریب Chronbach's alpha بررسی شد. تحلیل عاملی اکتشافی برای کاهش تعداد آیتم‌ها به کار رفت. یافته‌ها: در ارزیابی محتوایی، 5 آیتم حذف گردید. در تحلیل همبستگی نیز، 24 آیتم که دارای همبستگی کمتر از 3/0 با همبستگی کل داشتند، حدف شدند. ضریب Chronbach's alpha برای 16 آیتم باقیمانده، برابر 92/0 (85/0-71/0) به دست آمد. در تحلیل عاملی اکتشافی سه زیر مقیاس قضاوت ذهنی، لباس حفاظتی و فعالیت جسمانی استخراج شد؛ به طوری که هر سه زیر مقیاس، در مجموع 7/67 درصد از واریانس را تبیین کردند. ثبات درونی بین زیر مقیاس‌ها بالا بود (91/0-73/0). نتیجه: نتایج تحلیل عاملی اکتشافی نشان داد که سه عامل اساسی در مقیاس HSSI با ثبات درونی بالا وجود دارد. پایایی و روایی سازه نشان داد که مقیاس HSSI برای غربال‌گری تنش گرمایی در ایران یک روش روا و مناسب است.

Title: ارتباط دانسیته انرژی رژیم غذایی با سندرم متابولیک در زنان

http://hsr.mui.ac.ir/index.php/jhsr/article/view/162

مقدمه: با وجود تأثیر زیاد رژیم غذایی بر سندرم متابولیک، داده‌های محدودی در زمینه ارتباط دانسیته انرژی رژیم غذایی با این سندرم وجود دارد. به علاوه، با توجه به خصوصیات ویژه رژیم غذایی کشورهای خاورمیانه، بررسی ارتباط دانسیته انرژی غذایی و سندرم متابولیک در این بخش از جهان جالب توجه خواهد بود. این مطالعه به منظور ارزیابی ارتباط دانسیته انرژی رژیم غذایی با شیوع سندرم متابولیک در زنان انجام شد. روش‌ها: دریافت معمول غذایی طی مطالعه مقطعی بر روی 486 نفر از زنان بزرگ‌سال تهرانی با استفاده از پرسش‌نامه بسامد خوراک ارزیابی شد. دانسیته انرژی غذایی از تقسیم کل انرژی دریافتی روزانه بر کل وزن غذاهای مصرفی (gr/d) گزارش شده توسط هر فرد (به استثنای نوشیدنی‌ها) محاسبه گردید. شاخص‌های تن‌سنجی اندازه‌گیری و سطح گلوکز ناشتای پلاسما، فراسنج‌های لیپیدی سرم و فشار خون ارزیابی شد. سندرم متابولیک بر طبق راهنمای Ш ATP تعریف گردید. یافته‌ها: میانگین دانسیته انرژی رژیم غذایی پس از تعدیل اثر انرژی kcal/g 35/0 ± 77/1 بود. پس از کنترل اثر عوامل مخدوش‌گر، افرادی که در بالاترین سهک دانسیته انرژی قرار داشتند، 80 درصد (15/3-17/1: CI 95 درصد؛ 80/1 :OR) شانس بیشتری جهت ابتلا به سندرم متابولیک داشتند. حتی پس از تعدیل اثر BMI نیز این ارتباط، معنی‌دار باقی ماند (96/2-10/1: CI 95 درصد؛ 54/1 :OR). همچنین دانسیته انرژی غذایی بالاتر به طور معنی‌داری با شانس بالاتری برای چاقی شکمی (نسبت شانس بالاترین سهک در مقابل پایین‌ترین سهک: 23/4؛ 18/7-51/2 :CI 95 درصد)، سطح بالای‌ تری گلسیرید سرمی (93/5-31/2؛ 55/3) و سطح پایین HDL-C سرم (84/2-13/1؛ 80/1) همراه بود. کنترل اثر BMI به میزان اندکی روابط را کمرنگ‌تر نمود، ولی باز هم تمامی روابط معنی‌دار بودند. ارتباط کلی معنی‌داری بین دانسیته انرژی غذایی بالاتر و خطر فشار خون بالا یا اختلال در تحمل گلوکز، قبل و یا بعد از تعدیل اثر عوامل مخدوش‌گر، مشاهده نشد. نتیجه‌گیری: بالا بودن دانسیته انرژی رژیم غذایی به طور معنی‌داری با خطر بیشتر برای سندرم متابولیک و اجزای آن همراه بود. مطالعات بیشتری جهت تمرکز بر کاهش دانسیته انرژی رژیم غذایی به عنوان یک استراتژی احتمالی برای پیش‌گیری و درمان سندرم متابولیک نیاز می‌باشد.

Title: بررسی سرواپیدمیولوژیک توکسوپلاسموز در زنان با و بدون سابقه سقط جنین در شهرستان ایلام

http://hsr.mui.ac.ir/index.php/jhsr/article/view/170

مقدمه: توکسوپلاسموز از بیماری‌های انگلی مشترک بین انسان و دام با گسترش جهانی است با خوردن گوشت نپخته یا کم پخته حاوی کیست و آب آلوده به کیست یا از طریق مادرزادی از مادری که عفونت اکتسابی در طی حاملگی داشته باشد انتقال می‌یابد.هدف از این مطالعه تعیین وضعیت سرواپیدمیولوژیک زنان با سابقه و بدون سابقه سقط جنین مکرر نسبت به عفونت توکسوپلاسموز در شهرستان ایلام می‌باشد. روش‌ها: در این مطالعه مورد– شاهدی 122 نفر از زنان مراجعه‌کننده به درمانگاه زنان بیمارستان مصطفی خمینی ایلام به روش نمونه‌گیری تصادفی ساده در دو گروه‌، مورد با سابقة سقط جنین 63 نفر (7/51 درصد) و شاهد بدون سابقه سقط جنین 59 نفر (3/48 درصد) از نظرتیترآنتی بادیIgG و IgM علیه توکسوپلاسموز به روش ELISA مورد بررسی قرار گرفتند . پرسش‌نامه‌ای جهت گردآوری کلیه اطلاعات مورد نیاز از هر گروه تکمیل گردید. د‌اده‌ها توسط نرم افزار SPSS و تست‌های مجذور کای، مان ویتنی و T استیودنت مورد تجزیه وتحلیل قرارگرفت . یافته‌ها‌: از نظر تیتر آنتی بادی IgG علیه توکسوپلاسما در گروه مورد 42نفر (7/66 درصد) مثبت و 21 نفر (3/73 درصد) منفی بودند و این نسبت در گروه شاهد 27 نفر (8/45 درصد) مثبت و 32 نفر (2/54 درصد) منفی بود. این تفاوت از نظر آماری با (02/0P<) معنی‌دار شد‌. از نظر تیتر IgM علیه توکسوپلاسما در گروه مورد 35نفر (6/55 درصد) مثبت و 28 نفر (4/44 درصد) منفی و نسبت به گروه شاهد 2نفر (4/3 درصد) مثبت و 57 نفر (6/96 درصد) منفی مشاهده گردید‌. این تفاوت نیز با (0001/0P<) معنی‌دار بود. بین تعداد دفعات سقط جنین و تیتر آنتی بادی با (001/0P<) برای IgM و 03/0P< برای IgG تفاوت آماری معنی‌داری وجود دارد. بین نحوة طبخ گوشت و تیتر آنتی‌بادی تفاوت آماری معنی‌دار نیز مشاهده شد ( 03/0P< برای IgG و 002/0P< برای IgM). نتیجه‌گیری‌:. روش‌های غربالگری سرولوژیک برای مدیریت و روش‌های پیشگیری از عفونت و آلودگی دوران بارداری همراه با آموزش بهداشت توصیه می‌شود.

Title: بررسی اثر رژیم غذایی غنی از MUFA بر شاخص‌های تن‌سنجی در زنان دارای اضافه وزن

http://hsr.mui.ac.ir/index.php/jhsr/article/view/118

مقدمه: با توجه به ارتباط بین چاقی و چاقی شکمی با بیماری‌های مزمن مختلف و همچنین تأثیر متفاوت چربی‌های رژیم غذایی بر شاخص‌های تن‌سنجی، هدف از مطالعه حاضر، بررسی اثر مصرف رژیم غذایی غنی از MUFA بر شاخص‌های تن‌سنجی در زنان دارای اضافه وزن (9/29 ≥ BMI ≥ 25) می‌باشد. روش‌ها: مطالعه حاضر در قالب یک کارآزمایی بالینی از نوع متقاطع بر روی 17 زن 50-20 ساله با میانگین نمایه توده بدنی kg/m2 0/2 ± 6/27 انجام شد. افراد مورد مطالعه به صورت تصادفی انتخاب شدند به مدت 6 هفته از رژیم غذایی غنی از MUFA (16 درصد MUFA و 8 درصد SFA) و رژیم غذایی شاهد (16 درصد SFA و 8 درصد MUFA) استفاده نمودند. درصد انرژی حاصل از سایر درشت مغذی‌ها (از جمله PUFA)، در طول دو دوره یکسان در نظر گرفته شد. در انتهای 6 هفته، دوره washout به مدت 2 هفته اجرا گردید و سپس جای گروه‌ها تعویض گردید. این دوره نیز به مدت 6 هفته اجرا شد. در ابتدا و انتهای هر دوره از مطالعه، ارزیابی‌های تن‌سنجی صورت گرفت. جهت ارزیابی‌های آماری از آزمون t-test مزدوج استفاده شد. یافته‌ها: طبق ارزیابی دریافت‌های غذایی افراد، درصد انرژي حاصل از MUFA در طي دوره رژيم غذایی غني از MUFA برابر با 13 درصد و در دوره شاهد برابر با 7 درصد به دست آمد، اين مقادير براي SFA به ترتيب برابر با 5/8 و 14 درصد حاصل شد. ميانگين تغييرات وزن بدن در دوره غنی از MUFA برابر با 9/2- کیلوگرم و در دوره شاهد برابر با 3/2- کیلوگرم است (4/0 = P). این تغییرات برای BMI (Body mass index) به ترتیب برابر با kg/m2 1/1- در مقابلkg/m2  93/0- (6/0 = P)، برای توده چربی برابر با 2- کیلوگرم در مقابل 4/1- کیلوگرم (2/0 = P)، برای توده فعال بدن برابر با 9/0- کیلوگرم در مقابل 9/0- کیلوگرم (98/0 = P)، برای دور کمر 2- سانتی‌متر در مقابل 5/1- سانتی‌متر (5/0 = P) و برای WHR برابر با 0031/0- در مقابل 0014/0- (8/0 = P) به دست آمد. نتیجه‌گیری: اگرچه يافته‌هاي اين مطالعه حاكي از عدم وجود تفاوت معني‌دار بين دو نوع رژيم غذايي غني از MUFA و رژيم غذايي شاهد در تأثير بر شاخص‌های تن‌سنجی را نشان می‌دهد ولي به علت اهمیت چاقی و چاقی شکمی بر سلامت، انجام بررسي‌هاي طولاني مدت‌تر با حجم نمونه بالا ضروري به نظر مي‌رسد.

Title: شیوع اختلالات اسکلتی عضلانی وآنالیز وضعیت ارگونومی کارگران درگیر با حمل دستی کالا در صنایع لبنیات

http://hsr.mui.ac.ir/index.php/jhsr/article/view/171

مقدمه: امروزه شیوع اختلالات اسکلتی عضلانی از عوامل نگران کننده بهداشت و سلامت در هر جامعه‌ای می‌باشد. یکی از اصلی‌ترین علت اختلالات اسکلتی عضلانی را می‌توان وضعیت و شرایط نامطلوب کاری نام برد. هدف از این مطالعه برآورد شیوع اختلالات اسکلتی عضلانی و آنالیز وضعیت‌ ‌ای بدنی در حین کار‌کارگران درگیر با حمل دستی کالا در صنعت لبنیات بود. روش‌ها: فعالیت‌های کاری‌ 50 نفر (26 نفر گروه 1و 24 نفر گروه 2) از کارگران شاغل مرد در صنایع پخش لبنیات در سال 1388 مورد مشاهده قرار گرفت. از پرسش‌نامه نوردیک (Nordic) جهت برآورد شیوع اختلالات اسکلتی عضلانی و از روش(Ovako Working posture Analyzing System) OWAS جهت آنالیز وضعیت‌های ارگونومی‌ کاری و اطلاعات با نرم افزارآماری SPSS تجزیه وتحلیل شد. یافته‌ها: یافته‌ها نشان می‌دهد شیوع وضعیت‌های بدنی آسیب زننده به سیستم اسکلتی عضلانی در این حرفه بالا است به گونه‌ای که43 نفر (86 درصد) از کارگران مورد بررسی از اختلالات کمر، 22 نفر (44 درصد) از اختلالات شانه،33 نفر (66 درصد) از اختلالات زانو و 14 نفر (28 درصد) از اختلال در مچ دست بیش از 24 ساعت در یک سال شکایت داشتند. به طور میانگین 82/22 درصد از وضعیت‌های ارگونومی کاری کارگران نیاز به اصلاح داشت (نمره‌های 3، 4 در مقیاس OWAS). نتیجه‌گیری: با توجه به این که وضعیت‌های ارگونومی نامطلوب و اختلالات اسکلتی عضلانی در کارگران مورد بررسی بالا بود، اجرای مداخلات ارگونومی (فنی و مدیریتی)، تدابیر پیشگیری وبرنامه‌های آموزشی نسبت به کارگران در این صنایع امری ضروری به نظر می‌رسد. واژه‌هاي‌ كليدي: وضعیت ارگونومی، حمل دستی کالا، صنعت لبنیات

Title: بررسی مقادیر TSPو PM10 و توصیف کیفیت هوا با تکیه بر شاخص AQI در هوای مناطق مركزی شهر تهران در سال 85-84

http://hsr.mui.ac.ir/index.php/jhsr/article/view/66

مقدمه: در عصر حاضر توسعه روز افزون جوامع شهری و افزایش فعالیت‌های اقتصادی و صنعتی بدون توجه به ارزیابی اثرات این فعالیت‌ها بر سلامت انسان به عنوان محور توسعه، آلودگی هوای شهرها و مخاطرات ناشی از انتشار آلاینده‌های مختلف را موجب گردیده است. از طرفی وضعیت بحرانی آلودگی هوای تهران بر کسی پوشیده نیست و با توجه به این که ذرات معلق به عنوان یکی از آلاینده‌های اصلی شهر تهران به شمار می‌رود، در این مطالعه غلظت ذرات معلق موجود در هوای آزاد شامل TSP و PM10 و همچنین توصیف کیفیت هوا با تکیه بر شاخص AQI بر اساس ماه‌های مختلف نمونه‌گیری مورد بررسی قرار گرفت. روش‌ها: این مطالعه از ابتدای دی‌ماه 84 تا پایان اردیبهشت 85 انجام شد. تعداد 50 نمونه برای TSP با استفاده از پمپ نمونه‌گیر با حجم زیاد و تعداد 50 نمونه برای PM10 با استفاده از دستگاه لیزری پایش ذرات مدل 107 برداشت شد. یافته‌ها: در طول دوره نمونه‌گیری از نظر آلاینده TSP، اسفند‌ماه 1384 با میانگین µg/m3 63/190 آلوده‌ترین ماه و فروردین 1385 با میانگین µg/m3 5/118 پاك‌ترین ماه بود. در طول دوره نمونه‌گیری از نظر آلاینده PM10، دی‌ماه 1384 با میانگین µg/m3 5/66 آلوده‌ترین ماه و فروردین 1385 با میانگین µg/m3 1/42 پاك‌ترین ماه گزارش گردید. نتیجه‌گیری: بر اساس شاخص کیفیت هوا (AQI) در طول دوره نمونه‌گیری، در 96 درصد از موارد آلاینده TSP کمتر از حد استاندارد (100 < AQI) و در 4 درصد از موارد آلاینده TSP بالاتر از حد استاندارد (100 > AQI) قرار داشت. بر اساس شاخص کیفیت هوا (AQI) در طول دوره نمونه‌گیری، در 98 درصد از موارد آلاینده PM10 در کمتر از حد استاندارد (100 < AQI) و در 2 درصد از موارد آلاینده PM10 بالاتر از حد استاندارد (100 > AQI) به دست آمد.

Title: ارتباط دریافت گوشت قرمز با سندروم متابولیک و غلظت CRP پلاسما در زنان

http://hsr.mui.ac.ir/index.php/jhsr/article/view/147

مقدمه: اگر چه مصرف گوشت قرمز با شیوع دیابت مرتبط است، اما برای نشان دادن وجود ارتباط بین دریافت گوشت قرمز و سندرم متابولیک و التهاب، اطلاعات کمی در دست است. هدف مطالعه حاضر مشخص کردن ارتباط بین دریافت گوشت قرمز، سندرم متابولیک و غلظت در گردش CRP به عنوان یک مقیاس التهاب سیستماتیک می‌‌باشد. روش‌ها: در این مطالعه مقطعی 482 زن معلم تهرانی با سن 60-40 سال مورد مطالعه قرار گرفتند و برای ارزیابی دریافت گوشت قرمز از FFQ استفاده شد. اندازه‌های آنتروپومتریک، فشار خون، گلوکز ناشتا خون، پروفایل‌های لیپیدی و غلظت CRP پلاسما مطابق روش‌های استاندارد ارزیابی شد. سندرم متابولیک به وسیله برنامه بین‌المللی آموزش کلسترول راهنمای ATP III پیشنهاد شده بود. یافته‌ها: میانگین و خطای معیار دریافت گوشت قرمز0/4 ± 9/45 گرم در روز  بود. بعد از کنترل آماری عوامل اغتشاش‌گر میانگین هندسی غلظت CRP پلاسما در بین هر یک از پنجک‌‌ها 46/1، 66/1، 73/1، 89/1 و 04/2 میلی‌گرم در لیتر بود.* در مدل‌های خام، افراد در پنجک بالای دریافت گوشت قرمز، ارتباط قوی‌تری را از نظر وجود سندرم متابولیک در مقایسه با افرادی که در پنجک پایین قرار داشتند، نشان دادند (01/0 > p-trend، 38/4-24/1CI: %95؛ 33/2: OR). این ارتباط بعد از تنظیم و تعدیل عوامل مغشوش‌گر نیز باقی ماند (01/0 < p-trend 01/4, 18/1:CI 15/2:OR). تعدیل CRP بر این ارتباط اثری نداشت (01/0>;p-trend 98/3- 16/1: ;CI 06/2: OR). نتیجه‌گیری: به طور کلی افزایش مصرف گوشت قرمز به صورت مقطعی با افزایش خطر سندرم متابولیک و التهاب ارتباط دارد. مطالعات آینده‌نگر برای تأیید این یافته مورد نیاز است.

Title: الگوهاي غذايي غالب، چاقي عمومي و چاقي شکمي در زنان معلم تهراني

http://hsr.mui.ac.ir/index.php/jhsr/article/view/27

مقدمه: مطالعه‌ حاضر با هدف شناسايي ارتباط الگوهاي غذايي غالب با چاقي عمومي و چاقي شكمي در زنان معلم تهراني انجام شد.  روش‌ها: در اين مطالعه مقطعي، 486 فرد 60-40 ساله از معلمان زن تهراني به روش نمونه‌گيري خوشه‌اي چند مرحله‌اي و به صورت تصادفي انتخاب شدند. دريافت‌هاي غذايي افراد با استفاده از يك پرسش‌نامه‌ نيمه كمي بسامد خوراك، براي يك سال ارزيابي گرديد. وزن و قد طبق دستورالعمل‌هاي استاندارد اندازه‌گيري و نمايه‌ توده‌ بدنی (Body mass index یا BMI) محاسبه شد. چاقي عمومي، به صورت BMI بزرگ‌تر از kg/m2 30 و چاقي شكمي به صورت دور كمر بزرگ‌تر از 88 سانتی‌متر تعريف شد. جهت شناسايي الگو‌هاي غذايي غالب، از روش تحليل عاملي استفاده شد. یافته‌ها: با استفاده از روش تحليل عاملي، سه الگوي غذايي غالب شامل الگوي غذايي سالم، الگوي غذايي غربي و الگوي غذايي سنتي در افراد مورد مطالعه شناسايي شد. پس از كنترل اثر عوامل مخدوش كننده، افرادي كه در بالاترين پنجك امتياز الگوي غذايي سالم قرار داشتند، در مقايسه با افراد پايين‏ترين پنجك، از شانس کمتري جهت ابتلا به چاقي عمومي (نسبت شانس 28/0 و 95 درصد؛ فاصله‌ اطمينان 14/0 تا 53/0) و چاقي شکمي (نسبت شانس 30/0؛ فاصله‌ اطمينان 16/0 تا 55/0) برخوردار بودند، در حالي که آن‌هايي که در بالاترين پنجک الگوي غذايي غربي قرار داشتند، از شانس بالاتري جهت ابتلا به چاقي عمومي (نسبت شانس 73/2؛ فاصله‌ اطمينان 46/1 تا 08/5) و چاقي شکمي (نسبت شانس 74/5؛ فاصله‌ اطمينان 99/2 تا 99/10) برخوردار بودند. کنترل اثر ساير متغير‌هاي مخدوش کننده باعث تضعيف اين ارتباط‌ها گرديد اما حتي پس از کنترل اثر انرژي دريافتي، اين ارتباط‌ها هم در مورد چاقي عمومي و هم در مورد چاقي شکمي معني‌دار بودند. هر چند که هيچ‌گونه ارتباط معني‌داري بين الگوي غذايي سنتي با چاقي عمومي مشاهده نشد، اما آن‌هايي که در پنجک سوم اين الگو بودند، چه قبل (نسبت شانس 15/2؛ فاصله‌ اطمينان 18/1 تا 90/3) و چه بعد از کنترل اثر متغيرهاي مخدوش کننده (نسبت شانس 08/2؛ فاصله‌ اطمينان 09/1 تا 65/3) از شانس بالاتري جهت ابتلا به چاقي شکمي برخوردار بودند. نتیجه‌گیری: يافته‌هاي اين مطالعه حاكي از ارتباط معني‏دار بين الگوهاي غذايي غالب با چاقي عمومي و چاقي شكمي در بين زنان معلم تهراني است. انجام مطالعات آينده‌نگر جهت تأييد اين يافته‌ها پيشنهاد مي‏شود.

Title: تأثیر آموزش جنسی بر بهبود رضایت زناشویی زوجین شهر اصفهان

http://hsr.mui.ac.ir/index.php/jhsr/article/view/97

مقدمه: رضايت زناشويي مهم‌ترين و اساسي‌ترين عامل جهت پايداري و دوام زندگي مشترك است. رضايت زناشويي زمانی وجود دارد كه وضعيت موجود در روابط زناشويي با وضعيت مورد انتظار منطبق باشد. در صورتي كه زوج‌ها بتوانند سطح رضايت را در زندگي زناشويي افزايش دهند، خانواده را از آسيب‌ها محفوظ مي‌دارند. هدف این مطالعه تأثیر آموزش جنسی بر افزایش رضایت زناشویی و ابعاد آن شامل سازگاری با شخصیت همسر، بهبود ارتباط، رابطه جنسی، حل تعارض،وایجاد نقش‌های مساوات طلبانه می‌باشد. روش‌ها: مطالعه نیمه تجربی با روش نمونه گیری تصادفی روی 30 زوج (60 نفر) مراجعه کننده به فرهنگ‌سرای خانواده در شهر اصفهان در دو گروه آزمایش و کنترل انجام شد. اعضای دو گروه قبل از شروع جلسات پیش آزمون را تکمیل کردند. سپس زوجین گروه آزمایش بعد از یک هفته در جلسات آموزش جنسی شرکت کردند. بعد از گذشت یک ماه، هر دو گروه به پس آزمون پاسخ دادند. گروه آزمایش در 6 جلسه یک‌ساعت و نیم شرکت کردند. ابزار گردآوری داده‌ها پرسش‌نامه کوتاه انریچ و مشخصات دموگرافیک بود. اطلاعات از طریق آمار توصیفی (میانگین، واریانس، انحراف استاندارد) و آمار استنباطی (تحلیل کوواریانس) با نرم افزار SPSS 16 تجزیه وتحلیل شد. یافته‌ها: نتایج پژوهش با آزمون تحلیل کوواریانس نشان از تأثیر آموزش جنسی در افزایش رضایت زناشویی (001/0>P، 78/118=F) و خرده مقیاس‌های رضایت از شخصیت همسر (001/0>P، 89/69=F)، بهبود ارتباط (001/0>P، 98/90=F)، رابطه جنسی (001/0>P، 87/51=F)، حل تعارض (001/0>P، 46/74=F) بود ولی در خرده مقیاس‌های اوقات فراغت (28/0=P، 54/7=F) و علائق به خویشان و دوستان (18/0=P، 35/2=F) تفاوت معنی‌داری دیده نشد. نتیجه‌گیری: رابطه جنسي زن و شوهر مهم‌ترين عامل تعيين‌كننده زندگي زناشويي است. آموزش جنسی با افزایش رضايت جنسي می‌تواند باعث افزایش در رضايت‌مندي و شادكامي كلي زناشويي گردد.

Title: مطالعات ميداني جهت بررسي پديده تغذيه‌گرايي در تالاب انزلي

http://hsr.mui.ac.ir/index.php/jhsr/article/view/73

مقدمه: پديده تغذيه‌گرايي به چالشي جدي براي بسياري از اكوسيستم‌هاي آبي طي سال‌هاي اخير تبديل شده است. پديده مذكور در تالاب انزلي كه به عنوان يكي از اكوسيستم‌هاي پراهميت از ابعاد گوناگون زيست‌محيطي و اقتصادي مطرح مي‌باشد، به دليل افزايش بيش از حد بار مواد مغذي ناشي از فعاليت‌هاي انساني، كشاورزي و صنعتي در حوزه آبريز آن‏، سلامت تالاب را با مخاطرات جدي روبرو ساخته است. بنابراين در اين تحقيق، مطالعات ميداني و نمونه‌برداري از نقاط مختلف تالاب صورت گرفت و سپس اطلاعات به دست آمده، تحليل گرديد و در نهايت وضعيت تالاب از نقطه‌نظر تغذيه‌گرايي آن، كه از مهم‌ترين شاخص‌هاي وضعيتي تالاب‌ها و درياچه‌ها مي‌باشد، مورد بررسي قرار گرفت. روش‌ها: در اين تحقيق علاوه بر انجام مطالعات ميداني، جهت تعيين وضعيت كيفي تالاب از نقطه‌نظر بارهاي مواد مغذي و محاسبه ميزان ورودي‌ها به تالاب، نتايج به دست آمده از سنجش پارامترهاي نيتروژن كل و فسفر كل از 21 ايستگاه منطقه مورد مطالعه، به سامانه سيستم اطلاعات جغرافيايي منتقل شد و از طريق مقايسه با استانداردهاي بين‌المللي، وضعيت تغذيه‌گرايي تالاب مشخص گرديد. يافته‌ها: در اين مطالعه مشخص گرديد كه چون تالاب نسبت به سال‌هاي گذشته، پذيرنده بارهاي بيشتري از مواد مغذي است، اغلب بخش‌هاي تالاب حاوي غلظت بالايي از نيتروژن و فسفر كل است. همچنين بررسي‌هاي بيشتر با استفاده از روش پيشنهادي نورنبرگ بر مبناي شاخص‌هاي نيتروژن و فسفر كل نشان داد كه وضعيت‌ فوق‌‌‌مغذي در تمامي نقاط تالاب حاكم مي‌باشد. نتيجه‌گيری: هر چند كه در اين تحقيق از روشي بسيار ساده با كمترين هزينه محاسباتي جهت تعيين وضعيت تغذيه‌گرايي در تالاب انزلي استفاده گرديد‏، اما نتايج به دست آمده از اين تحقيق با نتايج تحقيقات قبلي انجام گرفته بر تالاب در اين راستا، تطابق خوبي از خود نشان مي‌دهد.

Title: اندازه‌گیری شاخص‌های فساد روغن مصرفی قنادی‌ها و اغذیه فروشی‌های شهرستان برخوار و میمه استان اصفهان در سال 1387

http://hsr.mui.ac.ir/index.php/jhsr/article/view/114

مقدمه: طی سرخ کردن زیاد روغن، اکسیداسیون با میزان بیشتری اتفاق می‌‌افتد که سبب تولید هیدروپراکسیدها و سپس ترکیبات فرار مانند آلدئیدها، کتون‌ها و اسیدهای کربوکسیلیک و سایر مواد شیمیایی نامطلوب می‌گردد. این مطالعه با هدف تعیین میزان عدد پراکسید، رانسیته، عدد اسیدیته در روغن‌های مصرفی در قنادی‌ها و سمبوسه فروشی‌های شهر برخوار و میمه انجام گردید. روش‌ها: تعداد 54 نمونه روغن از کارگاه‌های قنادی و سمبوسه فروشی در شهرستان برخوار و میمه به صورت تصادفی جمع‌آوری شد. آزمایش‌های تعیین عدد پراکسید، رانسیدیته و شاخص اسیدیته بر روی 5 گرم روغن انجام شد که به ترتیب میزان هیدروپراکسیدها، آلدئیدها و اسیدهای آزاد، طی مراحل اکسیداسیون اندازه‌گیری و مشخص شد. یافته‌ها: آزمایشات نشان داد روغن ها از نظر عدد اسیدی، سالم و فقط 4/7% از آنها فاسد بودند این یافته ها حاکی از آن است که 87% از روغن های قنادی و سمبوسه فروشی ها از نظر عدد پراکسید قابل مصرف می باشند. نتیجه‌گیری: نحوه استفاده روغن اغذیه فروشی و قنادی‌ها در شهرستان برخوار و میمه نامطلوب بوده است که ممکن است محتوی مواد سمی باشند و سلامت مصرف کنندگان را تهدید کنند. بنابراین مسؤولان مربوط علاوه بر توجه به تهیه و توزیع روغن‌های مناسب برای پخت، جهت رعایت ضوابط و مقررات بهداشتی باید بر مراکز تهیه و توزیع غذاهای آماده و قنادی‌ها کنترل دقیق داشته باشند و آموزش‌های لازم ارایه شود.

Title: تأثير آموزش مبتني بر الگوي اعتقاد بهداشتي بر ارتقای رفتارهای تغذیه‌ای پیشگیری کننده از پوكي استخوان در دانش آموزان دختر مقطع دوم راهنمايي اصفهان

http://hsr.mui.ac.ir/index.php/jhsr/article/view/100

مقدمه: بدون ترديد، تغذیه صحیح در پيشگيري از ابتلا به بسياري از بيماري‌ها، به ویژه پوكي استخوان و افزايش كارآيي فكري و فيزيكي نيروي كار، نقش مهمي دارد. نظر به اهميت آموزش تغذیه به کودکان در سنين مدرسه، اين پژوهش با هدف تعيين تأثير برنامه آموزش بهداشت مبتني بر الگوي اعتقاد بهداشتي بر آگاهي، نگرش و عملکرد دانش آموزان دختر مقطع راهنمايي اصفهان در خصوص ارتقای رفتارهای تغذیه‌ای پيشگيري كننده از پوكي استخوان انجام گرديد. روش‌ها: مطالعه تجربی بر روي 130 دانش آموز دختر مقطع دوم راهنمايي اصفهان درسال 89- 1388 به صورت تصادفي در 2 گروه آزمون (66 نفر) و شاهد (64 نفر) به روش نمونه‌گیری چند مرحله‌ای انجام گرفت اطلاعات، پرسش‌نامه استاندارد مبتني بر الگوي اعتقاد بهداشتي و پرسش‌نامه FFQ (بسامد مصرف مواد غذايي) بود، پرسش‌نامه استاندارد الگوي اعتقاد بهداشتي در سه زمان قبل، همزمان و دو ماه بعد از آموزش تکميل گرديد و پرسش‌نامه FFQ در دو زمان قبل و 2 ماه بعد توسط دانش آموزان تکميل گرديد، بعد از انجام پيش آزمون، مداخله آموزشي در طي 4 جلسه آموزشي به مدت 4 هفته براي گروه آزمون انجام گرفت، اطلاعات‌ با نرم افزار SPSS 18 و آزمون‌هاي آماري مورد تجزيه و تحليل قرار گرفت. یافته‌ها: دو گروه از نظر متغيرهاي دموگرافيكي تفاوت معني‌دار با هم نداشتند. قبل از مداخله تفاوت معني‌داري بين ميانگين نمرات سازه‌هاي مختلف اين الگو در دو گروه مشاهده نشد (05/0<p). بعد از مداخله تفاوت معني‌داري در ميانگين نمرات آگاهي، حساسيت درک شده، شدت درک شده، منافع درک شده و موانع درک شده و عملكرد بين دو گروه آزمون و شاهد مشاهده شد (001/0p<). نتيجه‌گيري‌:‌ نتایج این مطالعه تأثير مثبتي روي الگوي تغذیه دانش آموزان داشته است و مداخله در افزايش و بهبود ميزان آگاهي، نگرش و عملكرد دانش آموزان مؤثر بوده است.

Title: بررسی و مقایسه دریافت انرژي و درشت مغذی‌ها در زنان ورزشکار آئروبیک در دو منطقه اجتماعي- اقتصادي مختلف شهر اصفهان در سال 1388

http://hsr.mui.ac.ir/index.php/jhsr/article/view/119

مقدمه: هدف از اين مطالعه، ارزيابي دريافت‌هاي غذايي زنان ورزشكار آئروبيك در دو منطقه اجتماعي- اقتصادي مختلف در شهر اصفهان است. روش‌ها: در اين مطالعه تحلیلی 99 زن در رنج سني 50-15 سال كه با نمونه‌گيري تصادفي انتخاب شدند، شركت كردند (50 زن در منطقه اقتصادي پايين و 49 زن در منطقه اقتصادي بالا). اطلاعات دموگرافيك با كمك پرسش‌نامه و شاخص‌هاي تن‌سنجي شامل قد، وزن و دور كمر با متر نواري و ترازو جمع آوري شدند. با تكميل پرسش‌نامه يادآمد 24 ساعته براي 2 روز متوالي، اطلاعات غذايي افراد مورد مطالعه به دست آمد. جهت آناليز داده‌ها از نرم‌افزار 4 Nutritionist و 18SPSS استفاده شد. يافته‌ها: میانگین دریافت انرژی در زنان بالا شهر 86/561 ± 19/1479 و در زنان پایین شهر 354/498 ± 68/1300 کیلوکالری به دست آمد که تفاوت معنی‌داری بین دریافت انرژی در این دو گروه وجود نداشت (098/0 = P). ميانگين دريافت پروتئين در منطقه اقتصادي پايين 85/5 ± 41/17 درصد و در منطقه اقصادي بالا 12/4 ± 94/17 درصد به دست آمد كه تفاوت معني‌داري بين اين دو وجود نداشت (606/0 = P). ميانگين دريافت كربوهيدرات در منطقه اقتصادي پايين 76/9 ± 85/61 و در منطقه اقصادي بالا 62/6 ± 48/54 به دست آمد که تفاوت بين ميانگين‌ها، معني‌دار است (001/0 P <) و ميانگين دريافت كربوهيدرات در منطقه اقتصادي پايين به طور قابل توجهي بالاتر از منطقه اقتصادي بالا می‌باشد. ميانگين دريافت چربي در منطقه اقتصادي پايين 24/8 ± 88/23 درصد و در منطقه اقصادي بالا 68/6 ± 07/30 درصد به دست آمد كه تفاوت بین دو گروه معني‌دار است (001/0 P <) و ميانگين دريافت چربي در منطقه اقتصادي بالا به مقدار قابل توجهي بيشتر از منطقه اقتصادي پايين می‌باشد. نتيجه‌گيري: این مطالعه نشان می‌دهد که دریافت چربی و کربوهیدرات در دو منطقه اجتماعي- اقتصادي مختلف شهر اصفهان متفاوت است که ممکن است به دلیل تفاوت در الگوهای غذایی منطقه باشد.

Title: بررسی جذب سطحی فاضلابهای حاوی فرمالدوئید بر روی موی انسان

http://hsr.mui.ac.ir/index.php/jhsr/article/view/117

مقدمه: یکی از ترکیبات آلاینده که می‌تواند در فاضلاب‌های صنعتی وجود داشته باشد، فرمالدئید است. جذب بیولوژیکی این ترکیب تا غلظت‌های کم به دلیل خاصیت ضد باکتری فرمالدئید بسیار مشکل است، ولی حذف آن توسط جاذب‌ها امکان پذیر است. هدف اصلی در این مطالعه بررسی جذب فرمالدئید به کمک یک جاذب طبیعی (موی انسان) است. موی انسان جاذبی کم هزینه می‌باشد و به سادگی توسط روشی نظیر خاکستر سازی قابل دفع می‌باشد. همچنین بررسی تأثیر پارامترهای مهم بر جذب این ماده توسط موی انسان نیز در این تحقیق مورد توجه قرار گرفت. روش‌ها: در این مطالعه در شرایط آزمایشگاهی فاضلاب آلوده به فرمالدئید با غلظت و حجم معین در تماس با موی انسان قرار گرفت. با کمک اندازه‌گیری مقدار اکسیژن خواهی شیمیایی (COD) فاضلاب قبل و بعد از تماس، درصد جذب فرمالدئید از محیط مشخص گردید. در این بررسی مقادیر بهینه pH، دما و زمان ماند نیز با کمک روش یک عامل در زمان مورد بررسی و مطالعه قرار گرفت. یافته‌ها: نتایج به دست آمده نشان داد که امکان جذب فرمالدئید توسط موی انسان وجود دارد. همچنین مشخص گردید که افزایش زمان ماند تا 15 ساعت می‌تواند منجر به افزایش راندمان جذب گردد. بهترین pH جهت جذب حداکثر فرمالدئید برابر با 7 بود و در نهایت افزایش دما نیز منجر به افزایش مقدار جذب فرمالدئید می‌گردید. نتیجه‌گیری: فرآیند جذب فرمالدئید توسط موی انسان از نوع جذب شیمیایی می‌باشد. در این تحقیق مشخص شد که با کمک موی انسان می‌توان در شرایط بهینه با راندمان به نسبت بالایی فرمالدئید موجود در محیط را از فاضلاب حذف نمود.

Title: تأثیر آموزش بر آگاهی، نگرش و عملکرد مادران در خصوص پیشگیری از تشنج ناشی از تب در کودکان

http://hsr.mui.ac.ir/index.php/jhsr/article/view/172

مقدمه: تشنج ناشي از تب یکی از شايع‌ترين مشکلات کودکان در سنین 5 ماهگی تا 6 سالگی می‌باشد. این مطالعه با هدف بررسی تأثیر آموزش بر آگاهی، نگرش و عملکرد مادران در خصوص پیشگیری از تشنج ناشی از تب در کودکان انجام شده است. روش‌ها: کارآزمایی بالینی روی 88 نفر از مادران دارای کودک زیر دو سال مراجعه کننده به مراکز بهداشتی و درمانی شهر اصفهان، با تقسیم تصادفی به دو گروه مداخله و کنترل انجام شد. داده‌ها با پرسش‌نامه‌ در دو زمان قبل و 1 ماه پس از مداخله آموزشی جمع‌آوری گردید. مداخله در 3 جلسه آموزشی به مدت 60 دقیقه انجام شد. اطلاعات با آزمون‌های آمار‌ی تجزیه وتحلیل گردید . یافته‌ها: میانگین سن مادران در گروه مداخله و کنترل به ترتیب 9/3±75/26 و 4±84/26 سال بود پس از مداخله آموزشی افزایش معنی‌داری در میانگین نمره آگاهی (001/0>P)، نگرش (04/0= P) و عملکرد (01/ 0 =P) مادران در گروه مداخله نسبت به زمان پیش از مداخله آموزشی ایجاد شد. همچنین تغییر معنی‌داری در میانگین متغیر‌های ذکر شده قبل و پس از مداخله آموزشی در گروه کنترل دیده نشد. نتیجه‌گیری: مداخله آموزشی با هدف کنترل تب به منظور پیشگیری از تشنج ناشی از تب در کودک زیر دو سال توانسته است با افزایش آگاهی و نگرش مادران سبب بهبود عملکرد آنان نیز شود.

Title: بررسی شدت ناراحتي‌هاي كمر با فاكتورهاي رواني- اجتماعی شغلی و عوامل ارگونوميگي در پرستاران بخش اورژانس

http://hsr.mui.ac.ir/index.php/jhsr/article/view/130

مقدمه: فاکتورهای مؤثر در ایجاد اختلالات اسکلتی- عضلانی ازجمله کمردرد از مباحث مهم دانش ارگونومی می‌باشد هدفِ این تحقیق بررسی رابطه شدت ناراحتی در ناحیه کمر با دو فاکتور عامل کمردرد شغلی- عوامل‌ خطر ‌ارگونومیکی و عوامل روانی- اجتماعی- که در پرستاران بخش اورژانس افزایش بالایی دارند، بود. روش‌ها: این مطالعه مقطعی بر روی 120 نفر از پرستاران بخش اورژانس سه بیمارستان در اصفهان انجام شد. در این تحقیق برای ارزیابی روزانه عواملِ شغلی روانی- اجتماعی، عوامل خطر ارگونومیکی و شدت ناراحتی کمر به ترتیب از پرسش‌نامه معتبر محتوای شغلی(Job Content Questionnaire)، چک‍‍‍ لیست شناسایی خطرات‌ارگونومیکی (NIOSH, 1994) و پرسش‌نامه نوردیک استفاده شد. سؤالات در پایان هر روز کاری به مدت پنج هفته از افراد پرسیده شدند. اطلاعات با آزمون آماری Spearman ، Mann-Whitney، اسمیرنوف کولوگروف و نرم افزارSPSS18/PASW  تجزیه وتحلیل گردید. یافته‌ها: نیاز کار، ماهیت شغل، تقابل اجتماعی با شدت ناراحتی کمر رابطه معنی‌داری داشتند (P-value<0.05). درحالی که میان آزادی تصمیم‌گیری با شدت ناراحتی کمر رابطه وجود نداشت. همچنین رابطه میان شدت ناراحتی کمر و عوامل خطر ارگونومیکی معنی‌دار بود (P-value<0.05).  نتیجه‌گیری: این مطالعه نشان داد که با کاهش تقابل اجتماعی و افزایش نیاز‌کاری، ماهیت شغل و عوامل‌ خطر ‌ارگونومیکی، شدت ناراحتی کمر در پرستاران بخش اورژانس افزایش می‌یابد. بنابراین به منظور کاهش شدت ناراحتی کمر در پرستاران، باید علاوه بر فاکتورهای ارگونومیکی، عوامل روانی اجتماعی را نیز مد‌نظر قرار داد.

Title: بررسی کارایی ماده منعقد کنننده PAClدر کاهش کدورت آب رودخانه شهر چای ارومیه و اثرات بهداشتی آن

http://hsr.mui.ac.ir/index.php/jhsr/article/view/40

مقدمه: امروزه با افزایش جمعیت، آب‌های سطحی از مهم‌ترین منابع مورد استفاده برای مصارف خانگی و صنعتی می‌باشند، كه جهت تصفیه متداول این آب‌ها از مواد منعقد كننده استفاده می‌شود. منبع تأمین آب آشامیدنی بخشی از شهر ارومیه، رودخانه شهر چای است. از این رو، هدف این تحقیق بررسی امکان کاربرد پلی آلومنیوم کلراید در تصفیه‌خانه آب شماره 1 ارومیه و بررسی اثرات بهداشتی آن بود. روش‌ها: در این مطالعه توصیفی– مقطعی، نمونه‌برداری در فصل بهار انجام شد و با استفاده از دستگاه جار‌تست، غلظت‌های مختلفی از ماده منعقد کننده PACl (Poly Aluminum Chloride) به نمونه‌ها افزوده شد، تا مناسب‌ترین غلظت آن به دست آید. در این آزمایشات همچنین مقادیر pH، کدورت، قلیاییت و مقادیر آلومینیوم باقیمانده نیز مورد آزمایش قرار گرفت. یافته‌ها: بر اساس نتایج حاصل از آزمایشات، غلظت آلومینیوم باقیمانده در محدوده (5 تا 20 mg/lit) PACl مصرفی، حداكثر mg/lit 29/0 و در محدوده مصرفی (20 تا mg/lit 40)، حداكثر 5/0 mg/lit بود. همچنین راندمان حذف کدورت در محدوده کمتر از NTU 100، در حدود 78 تا 98 درصد و در محدوده بیشتر از NTU 100، بالاتر از 99 درصد به دست آمد؛ به طوری که با افزایش کدورت، حذف آن بهتر صورت گرفته است. نتیجه‌گیری: به دلیل غلظت کم مورد نیاز PACl و در نتیجه تولید اندک لجن، مصرف قلیاییت، کم و کاهش اندک pH، کاربرد PACl اقتصادی بوده، از نظر بهداشتی نیز در غلظت‌های كمتر از 20 mg/lit، آلومینیوم باقیمانده كمتری داشت، ولی در غلظت‌های بالاتر از 20 mg/lit آلومینیوم باقیمانده ایجاد کرده، از لحاظ بهداشتی و ارتباط مشکوک آن با بیماری آلزایمر، نامناسب می‌باشد.

Title: بررسی همه‌‌گير‌شناسي حيوان گزيدگي در شهرستان آق قلا

http://hsr.mui.ac.ir/index.php/jhsr/article/view/132

مقدمه: حيوان گزيدگي يكي از معضلات و مشكلات بهداشت عمومي است كه بالقوه خطر ايجاد بيماري هاري را دارد. این پژوهش با همه‌گير‌شناسي حيوان گزيدگي در شهرستان آق قلا در طي سال‌هاي 1388-1379 انجام گرفت. روش‌ها: پژوهش حاضر توصیفی از نوع مقطعی می‌باشد. تعداد13142 موردحيوان گزيدگي ثبت شده در مركز درمان هاري شهرستان آق قلا به روش سرشماري وارد مطالعه شدند. ابزار گردآوري اطلاعات از طريق دفاتر ثبت مشخصات افراد مراجعه‌كننده به مركز هاري شهرستان آق قلا بود. داده‌های جمع‌آوری شده از طريق آمارتوصیفی به صورت توزیع فراوانی، درصد، میانگین و انحراف معیار و آزمون کا‌ی اسکوئر مورد تجزيه و تحليل قرار گرفت. یافته‌ها: تعداد 13142 مورد حيوان گزيده ثبت شده 9479 نفر (1/72درصد) مرد و 3663 نفر (9/27 درصد) زن بودند. اكثريت آنان (1/72 درصد) مرد بودند. ميانگين و انحراف معيار مصدومين 8/17±0/25 سال و اكثريت آنان (84 درصد) ساكن روستا بود. اكثريت موارد گزش باتعداد 12893(8/97 درصد) توسط سگ بوده و 6/69 درصد موارد در پا اتفاق افتاده بود. از نظر شغل دانش‌آموزان با 9/28 درصد بيشترين فراواني را داشت. بيشترين فراواني گزش در فصل بهار بود (8/28 درصد). ميانگين بروز 1222 در يك‌صد‌هزار‌نفر محاسبه گرديد. بيشترين ميزان بروز با 1678 در يك‌صدهزارنفر در سال 1383 و كمترين آن با 1005 در يك‌صدهزار‌نفر مربوط به سال 1388 بود.بین فصل و تعداد گزش ارتباط معنی‌داری وجود داشت. (P<0.05). نتیجه‌گیری: ميزان بروز حيوان گزيدگي در شهرستان آق قلا نسبت به ديگر مطالعات انجام شده در نقاط مختلف كشور بسيار بالا است. با توجه به صرف هزينه‌هاي زیاد ناشي از تزريق واكسيناسيون و سرم ضدهاري اقدامات لازم جهت كاهش اين معضل ضروري به‌ نظر مي‌رسد. واژ‌ه های كليدي: همه‌گیرشناسی، بروز، هاري، حيوان گزيدگي، آق قلا

Title: بررسی وضعیت دریافت انرژی از ریز مغذی‌ها و ارتباط آن با شاخص‌های تن سنجی در جانبازان اصفهان

http://hsr.mui.ac.ir/index.php/jhsr/article/view/151

مقدمه: مطالعات گوناگون نشان دادند که دریافت ریز مغذی‌های مختلف در جانبازان جنگ تحمیلی متفاوت با مقدار توصیه شده روزانه می‌باشد. در نتیجه جانبازان در معرض خطر سوء‌تغذیه قرار دارند. روش‌ها: این تحقیق یک مطالعه توصیفی- تحلیلی از نوع مقطعی بود. افراد مورد بررسی 106 نفر و جمعیت مورد مطالعه جانبازان با درصد جانبازی بالای 10 درصد شهر اصفهان بود. جمع‌آوری اطلاعات با استفاده از پرسش‌نامه‌های فرم اطلاعات عمومی و یادآمد 2 روزه خوراک انجام شد. بررسی داده‌ها با نرم‌افزار تغذیه‌ای 4N و نرم‌افزار آماری 10SPSS انجام شد. یافته‌ها: در این مقاله به بررسی میانگین دریافت ریز مغذی‌ها و شاخص‌های تن سنجی در افراد جانباز پرداخته شد. همچنین مقایسه میانگین دریافت ریز مغذی با مقادیر توصیه شده (RDA) و مقایسه میانگین شاخص‌های تن سنجی با مقادیر استاندارد انجام شد. تعیین ارتباط بین مقدار دریافت ریز مغذی‌ها با شاخص‌های تن سنجی نیز انجام شد. نتیجه‌گیری: با توجه به یافته‌های مطالعه دریافتیم که الگوی دریافت غذایی جانبازان نیاز به اصلاح دارد. دریافت برخی از ریز مغذی‌ها کمتر از میزان توصیه شده بود با توجه به یافته‌های مربوط به شاخص‌های تن سنجی و ترکیب بدن در این گروه که نشان می‌دهد میزان چربی کل بیش از حد نرمال است، مشخص می‌شود که فعالیت بدنی منظم نقش مهمی برای ارتقای سلامتی این افراد دارد.

Title: فراواني برخي عوامل مؤثر در انتخاب نوع زايمان در شهرستان خميني‌شهر، استان اصفهان، 1387

http://hsr.mui.ac.ir/index.php/jhsr/article/view/58

مقدمه: در زايمان‌هاي سزارين، مرگ و مير مادر به طور چشمگيري افزايش مي‌يابد. علل اصلي مرگ شامل آندوميومتريت، خونريزي، عفونت دستگاه ادراري و ترومبوآمبوليسم می‌باشد. ميزان شيوع سزارين در اصفهان در سال 1387 به 4/52 درصد و در خميني‌شهر به 54 درصد رسيده است و ‌اين در حالي است كه سازمان جهانی بهداشت براي سال 2010 هدف را 15 درصد بيان نموده است. هدف از اين مطالعه تعيين عوامل مؤثر بر انتخاب نوع زايمان مي‌باشد. روش‌ها: پژوهش به صورت مقطعي در بين 190 نفر از زناني كه در سه ماهه نخست سال 1387 زايمان كرده بودند صورت گرفت. اطلاعات از طريق پرسش‌نامه و با انجام مصاحبه تكميل گرديد و از آزمون‌هاي  t-testمستقل، كاي اسكویر، آزمون همبستگي اسپيـرمـن و همبستـگي پيـرسون استفـاده شد و 05/0 ‌< P معني‌دار تلقي گرديد. یافته‌ها: نتايج نشان دادند که ميانگين و انحراف معيار نمره آگاهي در زنان با زايمان طبيعي3/2 ± 71 و در زنان با سزارين 2 ± 6/62 و ميانگين و انحراف معيار نمره نگرش در زنان با زايمان طبيعي 1 ± 5/51 و در سزارين 8 ± 7/45 است، همچنین آزمون t-test تفاوت بين دو گروه را معني‌دار نشان داد. توزيع فراواني عوامل مؤثر در انتخاب نوع زايمان نيز بر حسب نوع زايمان تفاوت معني‌دار نشان داد. نتيجه‌گيري: علاوه بر آگاهي و نگرش، فاكتورهاي ديگري نيز در انتخاب نوع زايمان مؤثر هستند كه از آن جمله مي‌توان پزشك، همسر، دوستان و اعتقادهای شخصي را نامبرد. بنابراین با افزايش ميزان آگاهي و بهبود نگرش مادران، مي‌توان در جهت كاهش ميزان سزارين گام برداشت.

Title: بررسی كیفیت میكروبی آبمیوه‌های تازه در آبمیوه فروشی‌های شهر ایلام در سال 1389

http://hsr.mui.ac.ir/index.php/jhsr/article/view/69

مقدمه: عدم رعایت اصول بهداشتی در هنگام نگه‌داری، انتقال، فرآوری و مصرف مواد غذایی می‌توانند سبب فساد زود هنگام و ایجاد ضررهای اقتصادی و نیز ایجاد بیماری‌های عفونی در مصرف کننده گردد. در این تحقیق آبمیوه‌های موجود در مراکز تهیه و توزیع شهر ایلام در سال 1389 از نظر وجود اشرشیاکلی و تعداد کلیفرم، باکتری هوازی، استافیلوکوکوس آرئوس، کپک و مخمر مورد بررسی قرار گرفت، تا زمینه‌ای جهت کنترل بهتر آلودگی‌ها فراهم گردد. روش‌ها: این بررسی یک مطالعه توصیفی- مقطعی بود. از حدود 30 آبمیوه فروشی شهر ایلام در نیم سال اول سال 1389 یک نمونه برداشت شد. حجم نمونه برداشتی 250 میلی‌لیتر بود، که در شرایط استریل و در دمای 4 درجه سانتی‌گراد در مدت کمتر از 6 ساعت به آزمایشگاه میکروبیولوژی منتقل گردید و بلافاصله مورد آزمایش قرار گرفتند. داده‌ها پس از گردآوری با نرم‌افزار Excel تجزیه و تحلیل گردید. یافته‌ها: نتایج این مطالعه نشان داد که حدود 60 درصد نمونه‌های مورد مطالعه به اشرشیاکلی، 33/73 درصد نمونه‌ها به کلیفرم، 66/46 درصد نمونه‌ها به باکتری‌های هوازی، 33/73 درصد نمونه‌ها به استافیلوکوکوس آرئوس و 33/63 درصد به مخمر آلوده بوده‌اند. هیچ گونه آلودگی از نظر کپک در نمونه‌ها مشاهده نشده است. نتیجه‌گیری: طبق استاندارد اداره نظارت بر مواد غذایی، آبمیوه‌های آماده مصرف در آبمیوه فروشی‌ها نبایستی اشرشیا‌کلی و استافیلوکوکوس آرئوس داشته باشند و حد مجاز کلیفرم، باکتری هوازی، کپک و مخمر به ترتیب 102، 105 × 5، 103، 104 در میلی‌لیتر آبمیوه می‌باشد. نتایج این تحقیق نشان داد که میزان آلودگی آبمیوه‌های مورد مطالعه بالا می‌باشد. از این رو نظارت و کنترل دقیق بر مواد غذایی بیش از پیش تأکید و پیشنهاد می‌گردد.

Title: تحلیل فضایی و سطح‌بندی بهداشتی استخرهای شنا

http://hsr.mui.ac.ir/index.php/jhsr/article/view/82

مقدمه: استخرهای شنا به دلیل استفاده مداوم و همچنین شرایط بهداشتی متفاوت افراد استفاده‌کننده ممکن است مکانی مناسبی برای انتقال انواع بیماری‌ها باشد. این موضوع از نگرانی‌های مهمی است که همیشه از نقطه نظر بهداشتی مطرح بوده است. روش‌ها: در این تحقیق توصیفی، نمونه‌گیری به صورت غیراحتمالی هدف‌دار انجام شد. مرکز بهداشتی و درمانی شماره یک اصفهان با 19 مرکز بهداشتی و درمانی و 416832 نفر جمعیت، در مجموع 19 استخر را تحت پوشش دارد. با بررسی‌های انجام شده، 16 استخر مورد بررسی قرار گرفتند. اطلاعات لازم به صورت میدانی و همچنین با استفاده از اطلاعات مستند شده در مرکز بهداشتی و درمانی فراهم شدند. یافته‌ها: استخرهای مورد بررسی 50 درصد دولتی و 50 درصد به صورت خصوصی بودند. استخرهای مورد بررسی 63 درصد سر پوشیده و 37 درصد رو باز بودند. از مجموع 16 استخر، 63 درصد دارای سیستم تصفیه می‌باشند و 37 درصد سیستم تصفیه آب ندارند. توزیع مکانی استخرها در محدوده مورد مطالعه توزیع مناسبی نیست و از مجموع 19 مرکز بهداشتی و درمانی، 9 مرکز استخری در محدوده آن‌ها وجود ندارد در حالی‌که این 9 مرکز، وسعتی بیش از 50 درصد و جمعیتی معادل 206743 نفر را دارا می‌باشند. یک از استخرها استاندارد لازم از نظر رعایت فاصله از بزرگراه را نداشت. نتیجه‌گیری: بررسی کامل در مورد شرایط بهداشتی و فضائی استخرها و برآورد تعداد استخر مورد نیاز مبتنی بر افراد، در محدوده مرکز بهداشتی شماره یک اصفهان ضروری می‌باشد. همچنین تهیه بانک اطلاعاتی کامل با استفاده از نرم افزار GIS که بتواند در اختیار محققین قرار گیرد، مورد نیاز است.

Title: مروری بر دستورالعمل‌ها و استانداردهای كیفی كمپوست

http://hsr.mui.ac.ir/index.php/jhsr/article/view/163

کمپوست یک روند تدریجی در طبیعت است که به وسیله آن مواد آلی با حضور هوا توسط میکروارگانیسم‌ها و قارچ‌ها تجزیه می‌شوند و حاصل آن کود با ارزشی است که می‌تواند در جاهای مختلف و حتی در باغچه‌های خانگی مورد استفاده قرار گیرد. به دلیل اهمیت کمپوست خانگی، از آن به عنوان ثروتی که دیده نمی‌شود، اشاره شده است. با توجه به اهمیت کمپوست به جا است که دستورالعمل‌ها و استانداردهای کمپوست مورد بررسی قرار گیرد. در این مقاله مروری بر دستورالعمل‌ها و استانداردهای كیفی كمپوست صورت گرفته است و به استانداردها و حد مجاز غلظت عناصر در کمپوست اشاره و مقایسه صورت گرفته است. رعایت استانداردها به منظور کنترل کیفیت محصول نهایی حاصل از فرایند تولید کمپوست، حایز اهمیت می‌باشد. تطابق پارامترهای کنترل کیفی کود نهایی با استانداردها، سبب می‌گردد که محصولی مناسب به بازار فروش عرضه گردد. ضمن این که از اثرات نامطلوب محصول بر محیط زیست، چرخه غذایی و غیره نیز جلوگیری می‌شود.
